# Supplementary material for: Do General Practitioners in a Visiting Medical Officer Arrangement Improve the Perceived Quality of Care of Rural and Remote Patients? A Qualitative Study in Australia
Source: Healthcare (Basel). 2022 Jun 4;10(6):1045. doi: 10.3390/healthcare10061045 (PMC9223112; doi:10.3390/healthcare10061045)
Supplement: Supplementary file 1 [file healthcare-10-01045-s001.zip › healthcare-1670721-supplementary.pdf]

Interview Guide for GP VMO

"An evaluation of the role of rural GP Visiting Medical Officers (GP-VMOs) on the quality of care and health outcomes in rural hospital of NSW"

Introduction

Thank you for your participation in this study. This interview is about your experiences of working as GP VMO in rural NSW, and your views on how GP VMOs can improve the quality of healthcare, patient safety and health outcomes of rural and remote patients. This interview will last for about 45 – 60 minutes. Do you have any questions before we proceed?

Socio-demographic characteristics

Leading question	Probing questions	Aims
Can I confirm your name and practice location please?	GP practice location?<br>Hospital name and location?	Understanding general information of the participant
Can I also confirm your date of birth or age please?	-	
Can I also please confirm if you are born in Australia?	If no, probe where?<br>When did you migrated to Australia?	Understanding social and training background of the participant
Do you trained as GP in Australia?	If no, probe where? Any issues or problems in adjusting with Australia's systems and culture?	
Can you tell me about your working history as GP VMO please?	Do you ever work as GP VMO previously? If yes, probe where, when and for how long?	Understanding previous experiences working as GP VMO
	With the current employment, how long have you been working as GP VMO?	

Motivation

Leading question	Probing questions	Aims
You have been doing this GP-VMO roles for quite some time now, can you please tell me what motivates you in pursuing this role?	Probing points:<br>(1) Financial incentives?<br>(2) Appreciation and support from hospital?<br>(3) Improving skills in emergency medicine or hospital care?<br>(4) Get more training?<br>(5) Love for the combined roles?<br>(6) Community respect?<br>(7) Township or community driven (helping local communities)?	Understanding motivation of rural GP in pursuing the GP-VMO role
What is your future aspiration in your medical career?	Probing questions:<br>(1) Do you intend to continue working as rural GP – VMO? If so, why and for how long?<br>(2) If not, what might be the key factors that discourage you in continuing your role as rural GP-VMO?	Understanding the intention of working in rural areas as GP VMO

Experiences working as rural GP-VMO

Leading question	Probing questions	Aims
How would you describe your overall experiences working as rural GP VMO?	(1) Do you like or enjoy or satisfy? Why or why not?	Contextualising the working situation and overall experiences of rural GP VMO
From your memory, what are the most satisfying or stressing events you ever dealt with so far?	Probing:<br>Explore the reasons why the participant pick these particular events!	
Do you ever experience any distress associated with your role as GP-VMO? If so, can you please tell me about it?	Probing:<br>(1) Physical distress?<br>(2) Psychological stress?<br>(3) Families or personal relationships being disrupted?<br>(4) Night shifts?	
	If so, how do you cope/manage these?	
What do you think about the workload of doing GP practice and visiting medical officer in a local hospital?	If increase, how so?<br>How does it affect your overall health, satisfaction or wellbeing?	
	How does being a rural GP VMO affect your primary care practice, hospital care you had provided or relationship with community/patients?	

Perspective on effects of rural GP VMO on quality of care and health outcomes

Leading question	Probing questions	Aims
In your opinion, what might be the effects (both positive or negative) of GP VMO on the quality of care and health outcome for rural communities?	Probing:<br>(1) For patients? (e.g. improve access to care, satisfaction, safety, trust to healthcare)<br>(2) For GP VMO? (e.g. satisfaction, improved skills, further training opportunities, clinical skills practice)<br>(3) For hospital? (e.g. reduce waiting time, reduce hospitalisation, effective triage, freed-up hospital for serious conditions, prescription rate, number of testing required, length of stay, continuity of care, efficiency)<br>(4) For communities or other social benefits? (e.g. appreciation from communities, closer to community so reduce social disruption, trust building)	Exploring effects of GP VMO on the quality of care and health outcomes of rural patients
Based on your personal experiences, how can rural GP VMO improve the quality of care and health outcomes of rural patients?	Probing:<br>(1) Bring social knowledge or understanding about the patient into hospital care to allow patient-centred care?	Understanding GP VMOs perspective on the roles of GP VMO in improving quality of care and health

	(2) Improving service delivery model and management plan by bringing personal relationship from primary care into hospital care?<br>(3) Making care closer to the community to minimise social disruption?<br>(4) Improving trust from rural communities to healthcare systems?<br><br>Note:<br>Please also probe other possible explanations/mechanisms for the improved quality or health outcomes!	outcomes of rural patients

Future improvements for GP VMO program

Leading question	Probing questions	Aims
In your opinion, what might be the key challenges in implementing GP VMO program in Australia?	Probing:<br>(1) Availability of GP in rural town?<br>(2) Lack of skills, competence or confidence of rural GP?<br>(3) Lack of opportunities?<br>(4) Low financial incentives?<br>(5) Low retention rates?<br>(6) Social circumstances living in rural town leading to limited GP practice and low retention?<br>(7) Lack of information or updated information?<br>(8) Demanding job and heavy workload?	Exploring challenges and recommendations to improve GP VMO program and quality of care provided to rural communities
	Based on your experiences, what can be done by government, hospital, GP and community to improve GP-VMO program?	
How can we improve the quality of care provided to rural communities?	-	

Closing

Thank you very much for your time and your participation in our study. Before we end the interview, is there any question you would like to ask?

Interview Guide for Patients

"An evaluation of the role of rural GP Visiting Medical Officers (GP-VMOs) on the quality of care and health outcomes in rural hospital of NSW"

Introduction

Thank you for your participation in this study. This interview is about your experiences of being treated by your GP in your most recent presentations to emergency unit of local hospital, and your views on how GP VMOs can improve the quality of healthcare, patient safety and health outcomes of rural and remote patients. This interview will last for about 45 – 60 minutes. Do you have any questions before we proceed?

Socio-demographic characteristics

Leading question	Probing questions	Aims
Can I please confirm your name please?	-	Understanding social contexts of the participant
If you don't mind, can I please confirm your age?	-	
Are you currently married?	NB:<br>Expected responses can be currently married, never married, divorced/widow/widower	
Are you currently working?	If yes, probe what kind of occupation, where and for how long?<br>If not, probe where is the main income come from?	
What is your highest educational qualification?	If tertiary education, probe from where?	
Can you please tell me about your current living arrangements?	Probe:<br>Living with core or extended families? Renting or own property? Main mode of transportation?	

Access to GP and hospital

Leading question	Probing questions	Aims
On average, how many times do you visit your GP in a year?	Probe:<br>What is the main reason for your GP visit?	Contextualising access to GP and social relations between GP and patient
How long (in minutes) does it take from your place to your GP practice?	Probe patient's perspective about this distance – is it consider far or not really by the patient?	
How would you describe your care relationships with your GP?	-	
What about the hospital, how long does it takes from your place to the local hospital?	Probe patient's perspective about this distance – is it consider far or not really by the patient?	Exploring potential access barriers to healthcare services experienced by the patient
Does anything restrict your ability to access your GP or local hospital?	Probe:<br>Transportation issue? Mobility issue? No carers? Financial issue? Does not like healthcare facilities or don't trust health providers?	

Experiences of accessing emergency unit

Leading question	Probing questions	Aims
Can I please confirm when was your most recent presentation to emergency unit of local hospital?	Note:<br>It does not have to be exact, but probe a rough timeline (less than 1 month, 1-3 months, 4-6 month or more than 6 months ago).<br><br>Probing:<br>1. How do you get there (self-driven, ambulance, driven by families/friends)?	Exploring reasons for the ED presentation
Can you tell us why you presented to the emergency department at the local hospital?	Probing:<br>1. What happened?<br>2. Was is for acute or ongoing health issues?<br>3. Have you ever admitted to the hospital or presented to the ED due to the same health issues?	
How long do you have to wait before ED staff assisted you?	Note:<br>It does not have to exact, but probe a rough waiting time: <30 minutes, 30 minutes -1 hour, 1-3 hours, >3 hours<br><br>Probe patient's perspective about this waiting time – is it consider quick, average or long by the patient?<br>1. Did you admit to the hospital (hospitalisation)? If yes for how long?<br>2. Did you need further referral to tertiary hospital? If yes, why and where?	Exploring waiting time and overall experiences in accessing the ED at the local hospital
In general, how would you describe your experiences in accessing emergency unit at the hospital?	Probe:<br>Overall satisfaction? If not happy, explore what is the reason?	

Perspectives and experiences on being treated by GP-VMO

Leading question	Probing questions	Aims
Can I please confirm that during your most recent presentation at the emergency unit you were treated by your GP?	Probe:<br>1. Do you know that GPs often work as medical officers/staff in the emergency department? If yes, how did you find out about this?<br>2. Do you know that your GP also work at the hospital emergency department before your ED presentation? If you do, how did you find about it?<br>3. Did you personally request to be seen or treated by your GP or it happened by chance?<br>4. If you personally requested it, what are the main reasons?	Exploring the general knowledge, information and experiences of treating by GP VMO

What are your thoughts about being treated by your GP in the emergency department?		Probing:<br>(5) For patients? (e.g. improve access to care, satisfaction, safety, trust to healthcare, reduce waiting time, reduce hospitalisation, effective triage, continuity of care)<br>(6) For communities or other social benefits? (e.g. closer to community so reduce social disruption, trust building)		
What is your opinion about the quality of care provided to you in the hospital?		Probe:<br>Do you think having a GP working as a medical staff in the emergency department is helpful in your circumstances? If so why and how, and if not why and how?		Understanding patient's perspective on the quality of care provided by GP VMO and hospital more broadly
How do you feel about being treated by a medical staff that you are familiar with?		Probe:<br>1. Were there any concerns, challenges or potential conflicts for you with this mode of care/service delivery?<br>2. Would you have preferred to be treated by someone else you were not familiar with?<br>3. Do you mind sharing some positive or negative experiences of being treated by your primary care GP in the emergency department?		
Should you need to access care in the future in an emergency department, who would prefer to deliver the care for you?		Note:<br>Explore the reason for any option made by the participant!		Understanding patient's preference
Based on your personal experiences, how can rural GP VMO improve the quality of care and health outcomes of rural patients?		Probing:<br>(5) Bring social knowledge or understanding about the patient into hospital care to allow patient-centred care?<br>(6) Improving service delivery model and management plan by bringing personal relationship from primary care into hospital care?<br>(7) Making care closer to the community to minimise social disruption?<br>(8) Improving trust from rural communities to healthcare systems?		Understanding patient's perspective on the roles of GP VMO in improving quality of care and health outcomes of rural patients

Closing

Thank you very much for your time and your participation in our study. Before we end the interview, is there any question you would like to ask?
